# Assessment of the Suitability of Elastomeric Bearings Modeling Using the Hyperelasticity and the Finite Element Method

**DOI:** 10.3390/ma14247665

**Published:** 2021-12-12

**Authors:** Marcin Daniel Gajewski, Mikołaj Miecznikowski

**Affiliations:** Faculty of Civil Engineering, Warsaw University of Technology, 00-637 Warsaw, Poland; mikolaj.miecznikowski.dokt@pw.edu.pl

**Keywords:** elastomeric bearings, hyperelasticity, neo-Hookean model, Yeoh model, inverse method analysis, finite element method

## Abstract

The paper presents modeling of bridge elastomeric bearings using large deformation theory and hyperelastic constitutive relations. In this work, the simplest neo-Hookean model was compared with the Yeoh model. The parameters of the models were determined from the elastomer uniaxial tensile test and then verified with the results from experimental bearing compression tests. For verification, bearing compression tests were modeled and executed using the finite element method (FEM) in ABAQUS software. Additionally, the parameters of the constitutive models were determined using the inverse analysis method, for which the simulation results were as close as possible to those recorded during the experimental tests. The overall assessment of the suitability of elastomer bearings modeling with neo-Hookean and Yeoh hyperelasticity models is presented in detail.

## 1. Introduction

Elastomeric bearings are increasingly used in civil engineering. They are used not only in bridge engineering as elements through which the span load is transferred to the bridge supports, but more and more often as vibration-damping elements in steel and composite steel-reinforced concrete structures [1]. Depending on the nature of the work, the bearings undergo deformations of various sizes. In structural engineering, it is assumed that the application of the theory of small displacements and strains is sufficient for engineering purposes. However, observing the behavior of elastomeric bridge bearings, it can be seen that this limitation is often not met, i.e., it is not possible to predict the rational and experiential response of the bearing modeled in this theory.

Suitable modeling of the behavior of elastomeric bearings requires the application of the large deformation theory and hyperelasticity as the minimal constitutive modeling. Elastomeric bearings are made of natural or synthetic rubber, and their reinforcement is made of thin steel sheets. In the case of typical loads of such a bearing, the rubber regions deform significantly, while the steel sheets work in the elastic range (stresses do not exceed the yield point). Bearings are used in structures (e.g., bridge) to enable design movements of this structure resulting, for example, from changing ambient temperature. An additional advantage is their vibration damping properties. In the first case, it is sufficient to take into account the elastic properties of the materials, while in the second case, it is also necessary to take into account the visco-elastic properties of the material [2,3,4,5].

This article deals with the first situation and in the case of elastomers we limit ourselves to the use of hyperelasticity. Constitutive relationships of hyperelasticity are not commonly used to solve boundary problems in structural engineering, although it is possible to indicate several areas where they are necessary, i.e., for modeling membrane covers, for modeling the behavior of axially compressed elements (buckling problem), in the case of expansion joints [6] and elastomeric bearings. In relation to the linear elasticity of isotropic materials, the material parameters occurring in hyperelasticity relations are not so intuitively understood by engineers and hence probably the reason why they are not widely spread. The simplest model of hyperelasticity is the neo-Hookean model in which there are one (assuming the incompressibility of the material) or two parameters (in the case of a compressible material). However, this model has significant limitations [3,4] and should not be used as a rational model for elastomeric materials.

It has been shown in [7] that very reliable results can be obtained using the Yeoh model. In general, all hyperelasticity models predict nonlinear relationships between the stress and strain states in individual experimental tests, including a uniaxial tension test, pure shear test, biaxial compression/tension test, etc. Herein lies the first problem, which is the lack of the entire set of experimental tests. In most typical laboratories, we mainly have access to the uniaxial tensile test and basically determine the parameters of the model on that basis. Determination of the material parameter on that basis may be conducted in a rational way, such as in works [8,9], or may lead to some nonphysical behavior in different stress states. In this study, the parameters of the neo-Hookean and Yeoh models will be determined on the basis of the uniaxial tensile test and then verified using the experimental test with the heterogeneous state of stress and strain in the form of the compression test of the entire elastomeric bearing. It should be emphasized that the proposed procedure for determining the material parameters of the hyperelasticity models based on the uniaxial tensile and bearing compression test does not in any way diminish the importance of other material tests, such as biaxial tension or simple shear tests. It is always worth using as many experimental tests as it is possible, because it always increases the suitability of the model.

In the presented analysis it was assumed that the constitutive relations of steel have a secondary influence on the bearing response in the compression test, as the steel reinforcement deforms only slightly, and the generalization of the isotropic elasticity model into large deformations by changing the stress tensor to the Kirchhoff tensor, and the small-strain tensor to the logarithm of the left Cauchy stretch tensor is sufficient in this case. Such formulation is available in ABAQUS after considering the NLGEOM option and leaving the constitutive model of elastoplasticity as for small deformations. Such formulation is not objective; however, according to the comments included in the ABAQUS program manual, it may be approved for use if the strains does not exceed 5%. In the case of analyzed elastomeric bearings reinforcing steel, this condition is fulfilled. The consequences of such assumptions are analyzed in detail in [10].

In order to use the test with inhomogeneous stress and strain states (bearing compression test) to verify the constitutive relationship, it is necessary to formulate the inverse optimization problem, leading to application of the finite element method. The reverse optimization problem will consist of selecting such a set of material parameters of the constitutive model for which the global bearing response will be as close as possible to that recorded in the experimental study [11]. Similar issues were dealt with in [12,13,14] in relation to the constitutive relations of elastic–plastic materials.

To summarize, the following original issues are addressed in the paper:

- The suitability of the neo-Hookean and Yeoh models for usage in elastomer bearings modeling are verified in experiment of bearing compression.
- The procedure for extending the material experimental basis with the test of elastomer bearing compression using the inverse method and finite element modeling is proposed.
- FEM modeling results for different constitutive models and different sets of parameters are estimated, giving an idea of the size of the error and its consequences.

## 2. Determination of the Hyperelasticity Parameters Based on the Uniaxial Tension Test

### 2.1. General Form of the Strain Energy Density Function for Incompressible Materials

For the modeling of elastomer materials the large deformation theory of hyperelastic materials was used, which is well elaborated in many fundamental text books [15,16,17,18]. The elastomers used for bridge bearings may be treated as incompressible materials. When a material is considered incompressible, i.e. one whose volume does not change under a given load, the strain energy density function (SEDF) is independent of the strain invariant associated with volumetric changes, i.e., J=detF where F is the deformation gradient tensor and det() is a determinant, i.e.,

(1) $$W=U\left({\bar{I}}_{1},\;{\bar{I}}_{2},\;J\right)\to W=W_{D}\left({\bar{I}}_{1},\;{\bar{I}}_{2}\right),$$

where W is the general form of SEDF, $U\left({\bar{I}}_{1},\;{\bar{I}}_{2},\;J\right)$ is the strain energy density as a function of two isochoric invariants and volumetric invariant and $W_{D}$ is strain energy density for incompressible material (J=1). In Equation (1), standard notation of the hyperelasticity theory was used, e.g., [15,19]. For the record:

(2) $${\bar{I}}_{1}=J^{-\frac{2}{3}}I_{1},\;{\bar{I}}_{2}=J^{-\frac{4}{3}}I_{2},$$

where *I*_1_ and *I*_2_ are strain invariants defined as:

(3) $$I_{1}=\mathrm{tr}C=\mathrm{tr}B,I_{2}=\frac{1}{2}\left[{\left(\mathrm{tr}C\right)}^{2}-\mathrm{tr}C^{2}\right]=\mathrm{trCof}C=\frac{1}{2}\left[{\left(\mathrm{tr}B\right)}^{2}-\mathrm{tr}B^{2}\right]=\mathrm{trCof}B.$$

In Equation (3) C and B tensors are Cauchy–Green’s right and left tensors, respectively, i.e., $C=F^{T}F$ and $B=FF^{T}$, tr() stands for trace and Cof() for cofactor operations.

The use of the hybrid approach allows considering the volumetric part of the stress state using the Lagrange multiplier p, which is a variable independent of the kinematics description [20,21,22,23,24]:

(4) $$L({\bar{I}}_{1},\;{\bar{I}}_{2},J)=W_{D}\left({\bar{I}}_{1},\;{\bar{I}}_{2}\right)-p\left(J-1\right).$$

The consequence of such approach is the inability to obtain a constitutive relation solely on the basis of the strain state so it must take the following form

(5) $$\sigma =-pI+\frac{2}{J}\left(\frac{\partial U}{\partial {\bar{I}}_{1}}{\bar{B}}_{D}+\frac{\partial U}{\partial {\bar{I}}_{2}}{\bar{B}}_{D}^{-1}\right)=-pI+{\bar{\beta }}_{1}{\bar{B}}_{D}+{\bar{\beta }}_{-1}{\bar{B}}_{D}^{-1},$$

where the following deviatoric tensors were introduced ${\bar{B}}_{D}=\bar{B}-\frac{1}{3}{\bar{I}}_{1}I$, ${\bar{B}}_{D}^{-1}={\bar{B}}^{-1}-\frac{1}{3}{\bar{I}}_{2}I$.

It should be remembered that: $\bar{B}=J^{-\frac{2}{3}}B$, ${\bar{I}}_{2}=\mathrm{tr}{\bar{B}}^{-1}$ and the invariants (2) are the same for the Cauchy–Green deformation tensors. Tensors $\bar{B}$ and $\bar{C}$ are called left and right Cauchy–Green isochoric deformation tensors. In the constitutive relation (5), a scalar p is a Lagrange multiplier corresponding to the incompressibility constraints of the material (in the form J=1) and has an interpretation of an average pressure. The value p can be determined by solving a specific boundary problem. In this case, in finite element method (FEM) it is necessary to use the so-called hybrid finite elements, cf. [22,23], or other formulations with penalty functions.

### 2.2. Results of Uniaxial Tension Test (UTT) for the Analyzed Bearings Material 

The basic and the easiest to perform experimental test for rubber is an uniaxial tension test. The purpose of the test is to determine a number of tensile strength properties. During the test, the force is recorded depending on the elongation of the measuring section located on the narrow part of the paddle-shaped sample, while maintaining the specified standard speed of moving the jaws of the strength testing machine, which allows us to eliminate the influence of the viscous properties of the material. Uniaxial tensile tests were carried out on previously cut and measured samples. The result of the UTT is the relationship between the average stress and elongation defined as follows:

(6) $$S_{1}=\frac{P_{1}}{A_{0}},\;\lambda_{1}=\frac{l}{l_{0}},$$

where:

$P_{1}$—force (load) acting on the sample-average value from 5 tests;

l—actual length under a given load $P_{1}$;

$A_{0},\;l_{0}$—cross-sectional area and length of the measuring base before loading.

In the UTT there is the only one nonzero component of the first Piola–Kirchhoff stress tensor $S_{1}$. This means that in the constitutive relation given in Equation (5), the stress tensor should be changed using the relationship $J\sigma =SF^{T}$ and the principal elongations (stretches) should be introduced (it is worth remembering that first Piola–Kirchhoff stress tensor is conjugated to the deformation gradient tensor, it is a nonsymmetric two point stress tensor referencing to the initial configuration, cf. [15,25]). The elongation in the tensile direction is measured in an experiment, while the elongations in the perpendicular directions can be determined on the basis of the local incompressibility constraints, which can be expressed by the following relationship:

(7) $$J=\det F=\lambda_{1}\lambda_{2}\lambda_{3}=1.$$

To determine the material parameters of the constitutive models, data from the UTT for one of the five tests were used, for which the tensile plot in the largest part of the run was closest to the median of all tests [26], carried out on samples of the material used for the production of the modeled and further investigated elastomeric bearings.

### 2.3. Determination of the Neo-Hookean Model Parameters on the Basis of UTT

The simplest and, at the same time, one of the most popular models for describing incompressible materials is the neo-Hookean material [7,25], the SEDF of which is in the form of a linear function with respect to ${\bar{I}}_{1}$. This is a special case of Rivlin’s classic proposition [7,15,25].

The SEDF of the neo-Hookean model for incompressible materials is presented as dependent only on the first pseudo-invariant of isochoric deformation:

(8) $$W=W_{D}\left({\bar{I}}_{1}\right)=C_{10}\left({\bar{I}}_{1}-3\right),$$

where: $\mu_{0}=2C_{10}$ may be interpreted as the initial shearing modulus (for incompressible material $\nu_{0}$ = 0.5):

(9) $$\mu_{0}=G_{0}=\frac{E_{0}}{2(1+\nu_{0})}=3E_{0}.$$

It is worth noting that the neo-Hookean model cannot be used for any deformation. It is assumed that it can be used for elongation in the uniaxial tensile test at the level of 40%. Depending on how it is implemented, it can also pose many problems in predicting the shear test. In order to avoid such problems, constitutive relationships should be objective, cf. [27,28,29]. In the case of the analyzed bearings, the average compression is at the level of 7%; however, locally the deformations significantly exceed this value, both in terms of compression, tension and shearing. 

For the correct determination of the only parameter of the neo-Hookean model, the UTT is sufficient [7,25]. For this purpose, the results obtained in the laboratory, in which a homogeneous strain field was obtained, are approximated by the first Piola–Kirchhoff stress functions determined from the elastic energy potential.

Below, in Equation (10), the relationships between the elongation components resulting from the assumption of incompressibility are described and the only nonzero component of the Piola–Kirchhoff stress tensor of the first type in the uniaxial tensile test is determined, cf. Equation (11):

(10) $${\bar{\lambda }}_{2}={\bar{\lambda }}_{3},\;{\bar{\lambda }}_{3}={\bar{\lambda }}_{1}{}^{-\frac{1}{2}},\;{\bar{I}}_{1}={\bar{\lambda }}_{1}^{2}+2{\bar{\lambda }}_{1}^{-1},\;$$

(11) $$S_{1}=\frac{\partial W\left({\bar{\lambda }}_{1}\right)}{\partial {\bar{\lambda }}_{1}}=\frac{\partial W\left({\bar{\lambda }}_{1}\right)}{\partial {\bar{I}}_{1}}\frac{\partial {\bar{I}}_{1}}{\partial {\bar{\lambda }}_{1}}=\left({\bar{\lambda }}_{1}-{\bar{\lambda }}_{1}^{-2}\right)\mu_{0}.$$

The constitutive relation determined in this way may be used for approximation of UTT experimental results. Bearing in mind the fact that the only material parameter enters an Equation (12) linearly, the least squares method may be used for its determination. The function $S_{1}\left({\bar{\lambda }}_{1}\right)$ was fitted to the data from the uniaxial tensile test as in Table 1 for elongations in the range ${\bar{\lambda }}_{1}\in \langle 1.0,\;2.4\rangle$ using the NonlinearModelFit function available in the WolframMathematica environment [30].

Initial shear modulus was obtained as equal to:

(12) $$\mu_{0}=1.6987\;(\mathrm{MPa})\to C_{10}=\frac{\mu_{0}}{2}=0.8494\;(\mathrm{MPa}).$$

### 2.4. Determination of Yeoh Model Parameters on the Basis of UTT 

In the case of the Yeoh model, the SEDF takes the following form [9,14]:

(13) $$W=W_{D}\left({\bar{I}}_{1}\right)=C_{10}\left({\bar{I}}_{1}-3\right)+C_{20}{\left({\bar{I}}_{1}-3\right)}^{2}+C_{30}{\left({\bar{I}}_{1}-3\right)}^{3}.$$

Thus, the formula for the $S_{1}$ component of the Piola–Kirchhoff tensor of the first kind in the UTT can be written as:

(14) $$S_{1}=\frac{\partial W\left({\bar{\lambda }}_{1}\right)}{\partial {\bar{\lambda }}_{1}}=\frac{\partial W\left({\bar{\lambda }}_{1}\right)}{\partial {\bar{I}}_{1}}\frac{\partial {\bar{I}}_{1}}{\partial {\bar{\lambda }}_{1}}=\left({\bar{\lambda }}_{1}-{\bar{\lambda }}_{1}^{-2}\right){\bar{\beta }}_{1},$$

where:

(15) $${\bar{\beta }}_{1}=2\frac{\partial W}{\partial {\bar{I}}_{1}}=a_{1}+a_{2}{\bar{I}}_{1}+a_{3}{\bar{I}}_{1}^{2}=a_{1}+a_{2}\left({\bar{\lambda }}_{1}^{2}+2{\bar{\lambda }}_{1}^{-1}\right)+a_{3}{\left({\bar{\lambda }}_{1}^{2}+2{\bar{\lambda }}_{1}^{-1}\right)}^{2},$$

where the following substitutions are effectively used:

(16) $$a_{1}=2\left(C_{10}-6C_{20}+27C_{30}\right),\;a_{2}=4\left(C_{20}-9C_{30}\right),\;a_{3}=6C_{30}$$

Using the NonlinearModelFit procedure again to determine the parameters of the approximation function $S_{1}\left({\bar{\lambda }}_{1}\right)$ (using the Yeoh model) for the test results as in Table 1, we have obtained:

(17) $$\{\begin{array}{c} a_{1}=0.884\;(\mathrm{MPa})\\ a_{2}=0.0895\;(\mathrm{MPa})\\ a_{3}=0.0138\;(\mathrm{MPa}) \end{array}\to \{\begin{array}{c} C_{10}=0.638\;(\mathrm{MPa})\\ C_{20}=0.0430\;(\mathrm{MPa})\\ C_{30}=0.00229\;(\mathrm{MPa}) \end{array}$$

## 3. FEM Model and Its Convergence

### 3.1. FEM Model

**Figure 1:**
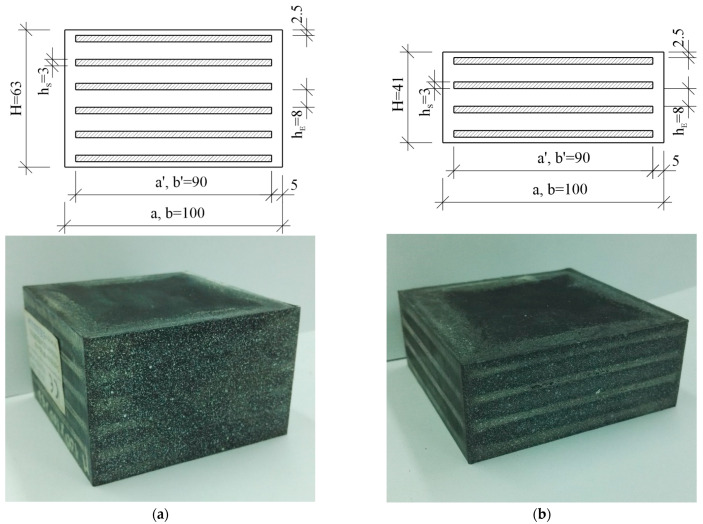
View of the considered bearings: (**a**) high bearing; (**b**) low bearing (dimensions in mm).

As mentioned in the introduction, constitutive models with material parameters determined on the basis of UTT will be verified in the compression tasks of two elastomeric bearings of different heights (marked as HB—high bearing and LB—low bearing) and the same cross-section. The bearings are made of natural rubber (NR). The external dimensions, number and geometry of individual layers of the considered bearings were determined on the basis of the dimensions provided by the manufacturer, our own measurements and on the basis of the bearing manufacturer’s catalog [31], cf. Figure 1 and Table 2.

Initially, for both bearings, a numerical experiment was carried out to compress the bearing by 10% of their height, which corresponds to displacements of 6.3 (mm) for the high bearing and 4.1 (mm) for the low one. Due to large deformations occurring during the experimental test, the NLGEOM option was used to account for geometric nonlinearity [18]. As part of the initial validation of the model, the elastomer was modeled using the parameters of the neo-Hookean model determined in Section 2.3. In the case of steel reinforcing elements, an elastic–plastic model with the Huber–Mises plasticity condition and isotropic strain hardening was adopted. The Young’s modulus was assumed to be 205 (GPa), the Poisson’s ratio was 0.3 and the yield point was 235 (MPa). It was assumed that after plasticizing the hardening modulus was equal to zero.

A three-dimensional bearing and the compression fixtures were modeled. The symmetry of the load and the geometry of the task allows us to consider one-eighth of the model, which significantly reduces the calculation time. For the bearing model, hybrid eight-node finite elements with linear shape functions and reduced integration C3D8RH were selected, and for the steel plate, hybrid ten-node finite elements with square shape functions C3D10H were selected.

**Figure 2:**
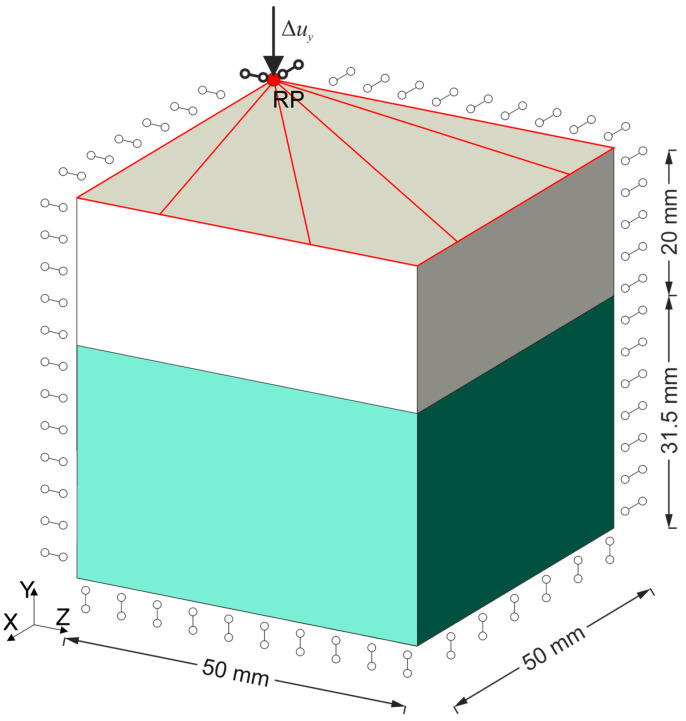
Implementation of the boundary conditions in the upper compression fixture using the MPC option [20,21] is marked in red while the symmetry conditions on the other three faces in the high bearing are shown symbolically.

The load was imposed by the displacement boundary conditions (Figure 2). To obtain the resultant force in a support, it is necessary to sum up the reactions in individual nodes. To avoid this process, a reference node has been introduced that is rigidly connected to all nodes lying on the top surface of fixture. The operation was performed using the MPC option available in the ABAQUS/Standard system package [20,21]. A detailed analysis of the boundary conditions during the compression, shear and rotation test of the bearing in the bridge structure is discussed in [7]. Symmetry conditions were assumed on the appropriate side surfaces of the considered region of the modeled bearing (Figure 2).

The tasks were solved using the Newton–Raphson algorithm, taking into account the automatic division of the step into increments as implemented in the ABAQUS/Standard program [22].

### 3.2. Convergence of the FEM Model

One of the most frequently overlooked elements that influences the correct assessment of the results obtained with the use of FEM is the influence of the mesh density. As a rule, a more detailed FEM mesh allows for more accurate results but requires a greater number of calculations performed by the computer processor, which extends the analysis time. The convergence analysis allows for the optimal selection of the finite element mesh in relation to the time of the analysis.

It is also worth noting that the entire FEM calculation process may not be convergent at all. The reasons for the lack of convergence of the solution may be incorrect selection of the size of the integration step, i.e., time step size or material instability. If the neo-Hookean material parameters are determined by fitting only with the uniaxial test, it is possible that in case of a more complex stress state the instability may be observed in the FEM simulations. Other reasons for the discrepancy which are reported in case of elastomers modeling is so called “volumetric locking” related to the incompressibility of the elastomers [22,24,32].

**Figure 3:**
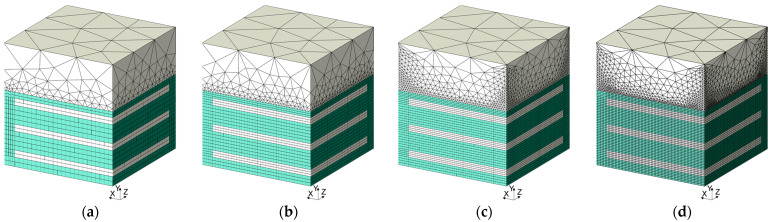
High bearing—finite element mesh: (**a**) M0; (**b**) M1; (**c**) M2; (**d**) M3.

It is therefore reasonable to carry out a convergence analysis for the bearing in question. Four meshes were adopted so that the thickness of the thickest rubber layer was divided into four finite elements for the coarse mesh (M0), six—for the medium mesh (M1), eight—for the detailed mesh (M2) and ten for the very detailed mesh (M3), cf. Figure 3.

For the proposed finite element meshes and the FEM model described above, a convergence analysis was performed. The table below summarizes the number of finite elements, the number of nodes, the analysis time and the final vertical support reaction determined for this task, see Table 3.

If we do not have an analytical solution to the problem, in most studies the error is determined between two successive refinements of the FEM mesh, until the previously assumed value of the relative difference between them is reached.

This paper presents a diagram of the load factor (LF) depending on the reciprocal of the number of nodes (Figure 3b) defined as:

(18) $$LF_{i}=R_{i}/R_{0},\;$$

where $R_{i}$ is a support reaction at the *i*-th mesh refinement, and $R_{0}$—support reaction obtained with an initial FEM mesh (in this case it is M0 mesh).

**Figure 4:**
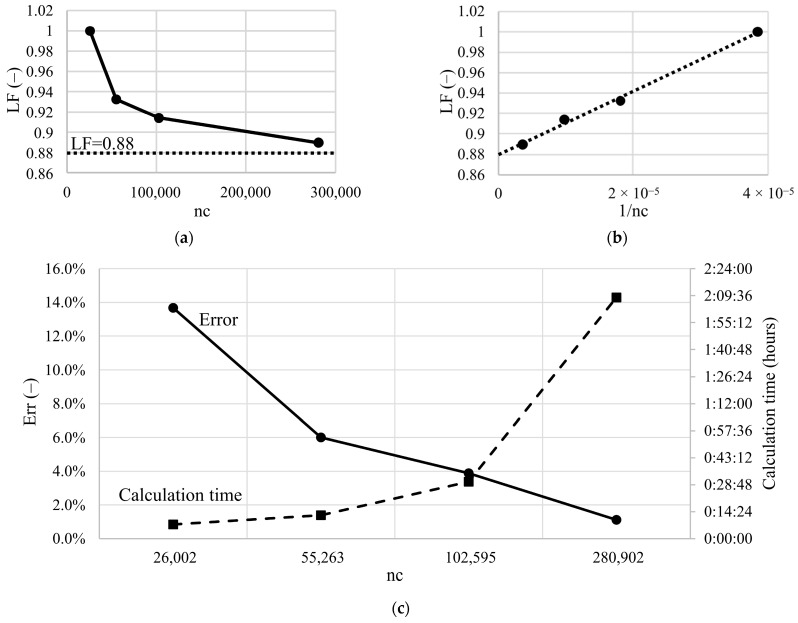
Convergence analysis—(**a**) normalized force depending on number of nodes; (**b**) normalized force depending on 1/number of nodes; (**c**) error and calculation time depending on number of nodes.

The points obtained in that way can usually be approximated linearly, which was also achieved this time. Based on the value of the intersection of the function determined with the axis of the load factor, we determine the support reaction in the case of the FEM model with an infinite number of nodes (nc — node counts) (if nc→∞ than 1/nc→0) (Figure 4b). The error of the support reaction with the assumed FEM mesh ($Err_{i}$) was defined as:

(19) $$Err_{i}=\frac{LF_{i}}{LF(1/\mathrm{nc}\to 0)}\cdot 100\%.\;$$

Figure 4c summarizes the calculation time and the error of the results with the assumed FEM mesh depending on the number of nodes [33].

In the case of the analyzed FEM meshes, a standard mesh verification procedure was also carried out using the tool implemented in ABAQUS CAE (“Verify mesh” tool). In all cases, mesh parameters such as aspect ratio, shape factor, angles on quad faces and tri faces were within normal (default) limits. Additionally, for all meshes, the comparison of the artificial strain energy to elastic recoverable strain energy was carried out, obtaining a ratio of no more than about 5% which proves that hourglassing is not a problem in analyzed cases. When selecting the FEM mesh, apart from the error value we obtain, we should also take into account the calculation time (Figure 4c). The propositions of the numerical algorithms which may be applied in hyperelasticity problems and shorten the calculation time are given for example in papers [34,35]. In this paper, a very detailed mesh (M3) will be used for individual simulations, while in the case of a large number of tasks, e.g., in inverse analysis, the calculations will be performed with the average mesh (M1).

## 4. Assessment of the Modeling Suitability

### 4.1. Elastomeric Bearing Compression Test

The compression tests (marked as HBCT—high bearing compression test and LBCT—low bearing compression test) were carried out on bearings supplied by a company that is a leading producer of elastomeric bearings on the local market. The tests were carried out in the universal testing machine INSTRON 8802. The measured values were the loading force and the displacement between the testing machine plates.

**Figure 5:**
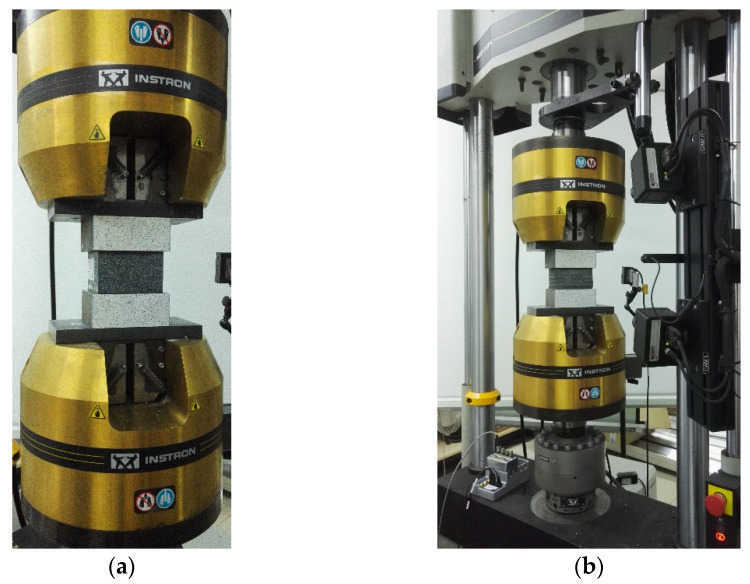
The elastomeric bearings placed in the testing machine (**a**) high bearing, (**b**) low bearing.

The pressure plates of the machine were attached centrally to the testing machine without the possibility of rotation. To both plates, steel spacers were attached (Figure 5).

The obtained force–displacement plots were scaled by the initial dimensions of the bearings as follows:

(20) $$S_{1}=\frac{P}{A_{0}},\;\lambda_{1}=\frac{H}{H_{0}},\;$$

where: 

P—value of the force loading the bearing; 

H—actual height of the bearing under a given load P;

$A_{0}$—the cross-sectional area of the unloaded bearing in the plane perpendicular to the load direction, $A_{0}=100\;({\mathrm{cm}}^{2})$ for both types of bearings;

$H_{0}$—height of the unloaded bearing (HB: $H_{0}=63\;(\mathrm{mm})$, LB: $H_{0}=41\;(\mathrm{mm})$).

### 4.2. Determination of the FEM Model Response with Material Parameters Determined in the UTT

**Figure 6:**
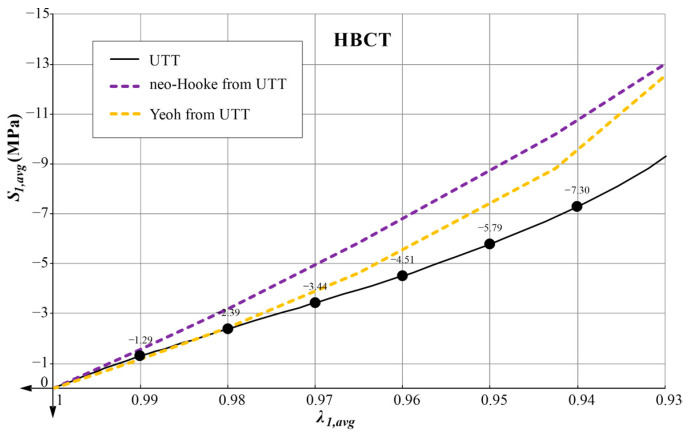
The response of high bearing, with the neo-Hookean and Yeoh model of rubber for material parameters determined on the basis of the UTT.

With the constitutive models of neo-Hookean and Yeoh together with the parameters determined from UTT and the FEM bearing models, we can solve the boundary problems of compressing high bearings. The obtained equilibrium paths are presented in Figure 6. The averaged stress is introduced on the vertical axis $S_{1,avg}=P/A_{0}$, and the averaged elongation in the direction of compression is on the horizontal axis. Comparing the obtained solutions with the results of the experiment, the consistency of all three graphs in the initial phase can be seen. After exceeding 1% of sample compression, the Yeoh model approximates the material behavior better than the neo-Hookean model. In the case of the Yeoh model, we have a compliance to about 2% compression of the sample. With further compression up to 7%, the predictions of both models are burdened with quite a significant error, but the error of the model with the Yeoh material is always smaller.

### 4.3. Comments on Determination of Hyperelasticity Parameters on the Basis of One Experimental Test

In hyperelasticity, taking into account the obtained nonlinear constitutive relations, it is inadvisable to determine material parameters on the basis of one experimental test. In the literature [36], it is proposed to determine material parameters on the basis of at least two of the three basic independent experimental tests, i.e., a uniaxial tensile test, a biaxial uniform tensile test and a pure shear test. Only the first of these tests is easy to perform in most laboratories. Due to the high degree of complexity of the reconstruction of the remaining homogeneous stress states, in order to determine the material parameters of elastomer bearings, in this paper a test supplementing UTT was proposed. This test carried out on the entire structural element is just an elastomer-bearing compression test.

## 5. Idea of Supplementing Material Tests with Testing of the Entire Bearing

**Figure 7:**
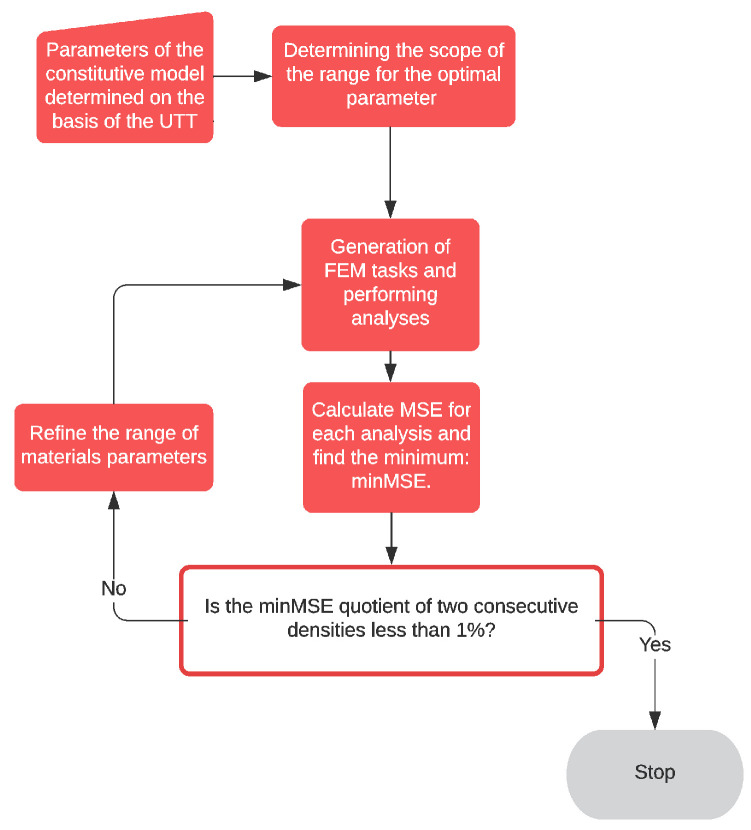
Inverse analysis algorithm—parametric study.

In order to determine material parameters, the inverse method analysis can be applied [37,38]. This analysis consists of solving a set of FEM analysis with boundary conditions reflecting the experimental test, in which the searched parameters of the constitutive model are changed in consecutive iterations. The starting point and the range of the search for optimal parameters were determined on the basis of the values determined in the UTT. The assumed range is divided into equal parts by the points at which calculations are performed. To solve a set of tasks with different material parameters, the ABAQUS/Standard package [20,21] provides the option of *Scripting parametric studies*. For the FEM model of the high bearing (HB) (Figure 2), a parameterized input file (identified with the extension. inp) had to be created, from which different variants of material parameters were generated using a script containing Python syntax (identified with the extension. psf). Due to the implementation from few to several tens’ FEM analysis, we decided to choose a model with a FEM M1 mesh for the optimization of the constitutive model. Then, for the parameters included in the inverse analysis, the normalized mean squared error of fitting the curves obtained in it to the experimental results was calculated. The error of fitting the analytically determined curve to its corresponding UTT results was calculated analogously. For the set of optimal parameters determined in this way, the search range is narrowed around them to half the distance between two adjacent values, and the calculations are performed once again. The calculations are stopped if the weighted sum of mean squared errors from both tests does not differ by more than 1% between successive iterations; see the algorithm shown in Figure 7.

In the next step, the correctness of the determined parameters of the constitutive models is verified by comparing it with the low bearing compression test (LBCT).

### 5.1. Formulating Inverse Method Analysis for the Neo-Hookean Model

Based on the UTT, the value of one coefficient of the neo-Hookean model (Section 2.3) was obtained equal to $C_{10}=0.8494\;(\mathrm{Mpa})$. After carrying out preliminary numerical analyses, it was noticed that the error in projecting the high bearing (HB) response curve decreased with the decrease in the parameter. Therefore, the range of material parameter for the inverse method analysis was adopted as follows:

(21) $$C_{10}=\{0.53,\;0.54,\;0.55,\;\ldots ,\;0.85\}\;(\mathrm{MPa}).$$

For each of the analyses, the normalized mean squared error of the curve fitting $S_{1,avg}(\lambda_{1,avg},\;C_{10})$ with the curve obtained from the experimental tests ${\bar{S}}_{1,avg}(\lambda_{1,avg})$ of the compression of the high bearing (HB) was calculated for n points on the curve where the error is calculated as:

(22) $$s_{HBCT}{}^{2}(C_{10})=\sum_{i=1}^{n=6}\frac{{\left(\frac{{\left(S_{1,avg}\right)}_{i}}{{\left({\bar{S}}_{1,avg}\right)}_{i}}-1\right)}^{2}}{n}.$$

The normalized mean squared error of fitting the curve given by the formula (11) to the curve obtained from the UTT was also calculated (Table 1) for n points on the curve where the error is defined as follows:

(23) $$s_{UTT}{}^{2}(C_{10})=\sum_{i=1}^{n=15}\frac{{\left(\frac{{\left(S_{1}\right)}_{i}}{{\left({\bar{S}}_{1}\right)}_{i}}-1\right)}^{2}}{n}.$$

The parameter was chosen for which the sum $s_{HBCT}^{2}(C_{10})+2\cdot s_{UTT}^{2}(C_{10})$ was the smallest:

(24) $$C_{10}=0.62\pm 0.005\;(\mathrm{MPa}).$$

For the normalized mean squared error of the UTT, the weight equal to two was assumed, bearing in mind the fact that this test was performed multiple times according to the standard. The HBCT test was performed only once without any special preparation, so as a consequence the unity weight was assumed in that case.

### 5.2. Formulating Inverse Method Analysis for the Yeoh Model

The determined values of Yeoh model parameters (compare Section 2.4):

(25) $$C_{10}=0.638\;(\mathrm{MPa}),\;C_{20}=0.0430\;(\mathrm{MPa}),C_{30}=0.00229\;(\mathrm{MPa})$$

were used as a reference to determine the model parameters using two material tests. It was assumed that the first parameter $C_{10}$, which is interpreted as half the initial shear modulus, will not change during the optimization process. In order to define the range for the two remaining parameters ($C_{20}$ and $C_{30}$), two values were chosen for each of them, one of which was greater and the other was smaller than those given in Equation (23), which limited the domain. For the first iteration, the limiting values were selected based on the literature [7,39] and the observation of the change in the UTT stress $S_{1}$ diagram when the parameters $C_{20}$ and $C_{30}$ were changed:

(26) $$C_{10}=0.638\;(\mathrm{MPa})\;\wedge C_{20}=\{-0.060,-0.035,-0.010,0.015,0.040,0.065,0.090,0.115,0.140\}\;(\mathrm{MPa})\;\wedge C_{30}=\{-0.050,-0.038,-0.025,-0.013,0,0.013,0.025,0.038,0.050\}\;(\mathrm{MPa}).$$

By multiplying the combinations of each of the given parameters, a set of 81 high bearing tasks was generated. For each task, the normalized mean squared error of fitting the normalized curve $S_{1,avg}(\lambda_{1,avg},\;C_{20},\;C_{30})$ against the curve ${\bar{S}}_{1,avg}(\lambda_{1,avg})$ obtained from the experimental high bearing compression tests was calculated for n points on the curve where the error is calculated as follows:

(27) $$s_{HBCT}{}^{2}(C_{20},C_{30})=\sum_{i=1}^{n=6}\frac{{\left(\frac{{\left(S_{1,avg}\right)}_{i}}{{\left({\bar{S}}_{1,avg}\right)}_{i}}-1\right)}^{2}}{n}.$$

For the set of parameters given in Equation (26), the normalized mean squared error of curve fitting was also calculated for the UTT. The uniaxial tensile curve $S_{1}(\lambda_{1},\;C_{20},\;C_{30})$ given by Equation (14) was fitted against the curve ${\bar{S}}_{1}$ obtained from UTT of elastomer (Table 1) for n points on the curve at which the error is calculated as follows:

(28) $$s_{UTT}{}^{2}(C_{20},C_{30})=\sum_{i=1}^{n=15}\frac{{\left(\frac{{\left(S_{1}\right)}_{i}}{{\left({\bar{S}}_{1}\right)}_{i}}-1\right)}^{2}}{n}.$$

For the normalized mean squared error of the UTT, the weight equal to two was assumed, then the values of the parameters for which the sum: $s_{HBCT}^{2}(C_{20},C_{30})+2\cdot s_{UTT}^{2}(C_{20},C_{30})$ was the smallest were selected, then the search range was refined between the two adjacent values of the selected numbers and the procedure was repeated until the weighted sum of mean squared errors from both tests obtained between successive iterations did not differ by more than 1%. The final result was obtained:

(29) $$C_{10}=0.638(\mathrm{MPa}),\;C_{20}=-0.0225\pm 0.002\;(\mathrm{MPa}),\;C_{30}=0.0161\pm 0.0008\;(\mathrm{MPa}).$$

## 6. Material Parameters Determined on the Basis of Inverse Analysis—Results and Their Verification

**Figure 8:**
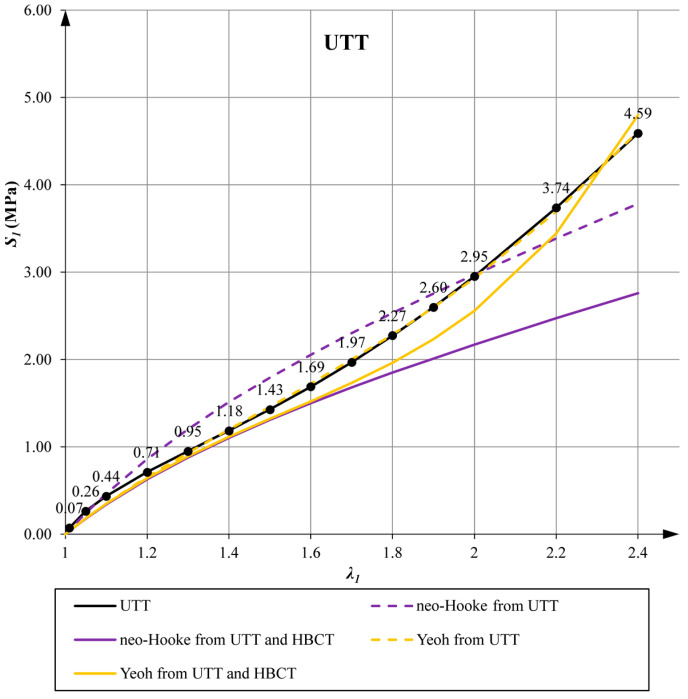
Comparative stress-elongation plot during UTT.

In Section 5, the neo-Hookean and Yeoh hyperelastic model parameters were determined from the UTT and HBCT. For the data determined in this way, the predictions of the models in UTT are presented in Figure 8. As expected, the accuracy of the representation of this test decreased when the additional HBCT was included. However, these plots show a clear advantage for the Yeoh model, which is able to predict the characteristic inflection point of the response function for $\lambda_{1}>1.4$.

**Figure 9:**
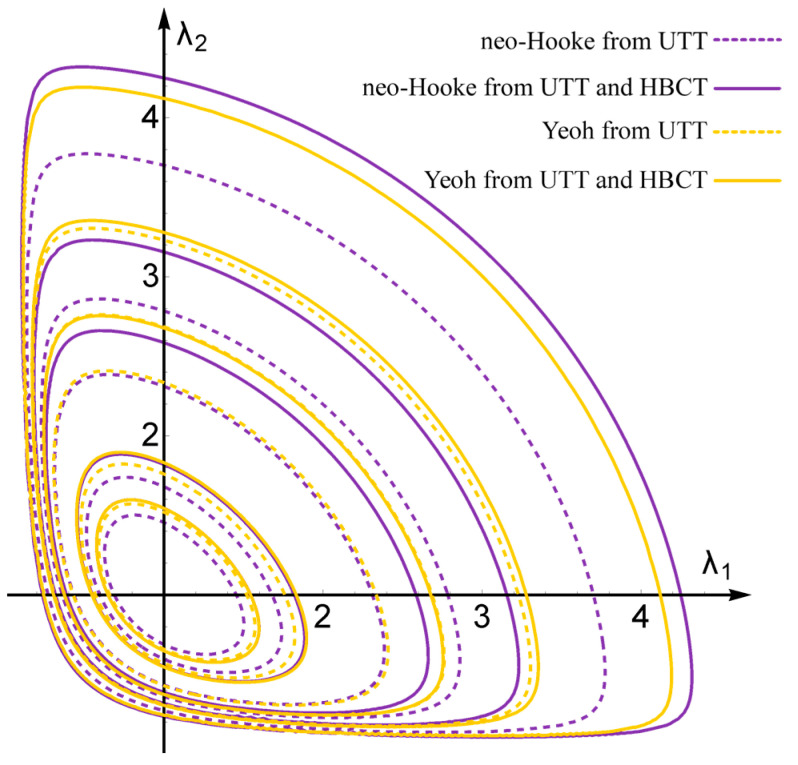
Contour plots of the SEDF for the data and models considered. The following contour lines correspond to the following SEDF values: 0.5, 1, 3, 5, 10 (MJ/m^3^).

Additionally, Figure 9 presents contour plots of the SEDF for the analyzed models with material parameters determined from either UTT or UTT + HBCT. These plots show a convexity of potentials in the range of large deformations (elongations up to values close to 4) and that the inclusion of HBCT in the data for determination of material parameters of constitutive models leads to faster increase in energy with increasing elongations.

**Figure 10:**
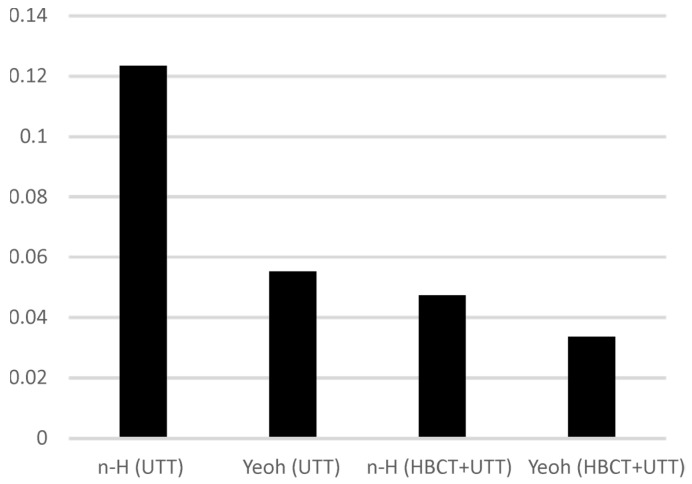
Normalized mean squared error for low bearing compression test with successive sets of material parameters.

In order to experimentally verify the results obtained, LBCT was performed for each of the four determined parameter sets and the normalized mean squared error of the curve fit was calculated. The bar chart in Figure 10 and Table 4 shows the results obtained.

**Figure 11:**
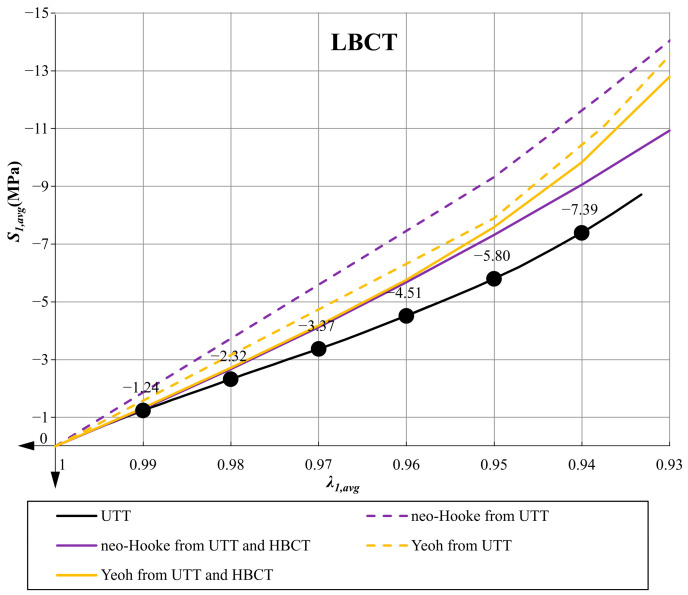
Comparative plot of the average pressure stress *S_*1*,avg_* as a function of the average elongation *λ_*1*,avg_* in case of LBCT.

**Figure 12:**
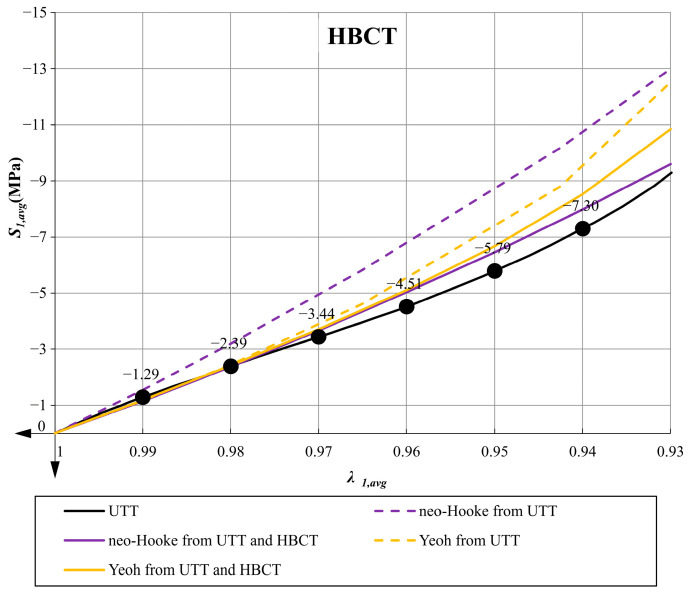
Comparative plot of average pressure stress *S_*1*,avg_* as a function of the average elongation *λ_*1*,avg_* in case of HBCT.

In Figure 11 and Figure 12 the plots of the average pressure stress as a function of the averaged elongation for low and high bearings are presented for all variants of determined parameters. 

## 7. Conclusions and Final Remarks

This paper proposes the procedure for modeling elastomeric bearings in the scope of the hyperelasticity theory. The main goal of this procedure is to increase the reliability of modeling by using constitutive models with parameters obtained on the basis of experimental tests with inhomogeneous deformation, strain and stress fields (in this case the HBCT and LBCT). Such a procedure requires the use of FEM and inverse analysis methods; however, it leads to a significant reduction in the error of projecting the bearing response determined in experimental tests. In the case of elastomeric materials, it is recommended to determine the parameters of the constitutive models of hyperelasticity on the basis of several experimental tests. Based on the calculations and comparisons discussed in this paper, it is clear that the use of UTT only is insufficient. It should also be emphasized that two constitutive models of neo-Hookean (the simplest possible) and Yeoh (more complicated, but correct from a theoretical point of view) were selected for the analysis, which show excellent stability and predictability in the large deformation theory, cf. Figure 9, showing the contour plot of the SEDF. Nevertheless, in the literature, there are models that are devoid of these features and in the large deformation theory their prediction may be irrational. In such cases, the determination of material parameters from just one test (UTT) can lead to nonphysical behavior in shear or biaxial tensile tests. In the modeled elastomeric bearings, the stress and deformation state are so inhomogeneous that locally we have situations similar to each of the above-mentioned tests, and even their coupling.

Based on the research presented in the article, the following conclusions may be formulated:

- The use of the Yeoh model, which satisfies all mathematical and physical requirements, always leads to better predictions of the material behavior in the uniaxial tensile test (UTT) and allows for a better representation of the behavior of the compressed elastomeric bearing than the use of the neo-Hookean model. In the case of the Yeoh model with material parameters determined from only one tensile test, the LBCT (Mean Squares Error) projecting error is at a comparable level as in the case of the neo-Hookean model with parameters determined on the basis of UTT and HBCT. Similar results were obtained in the work [40] for PLA materials in tensile tests at different temperatures. Uniaxial tensile tests and comparisons of these two material models were also performed in [8].
- Comparing the graphs in Figure 8 (UTT) with those in Figure 11 (LBCT) and Figure 12 (HBCT), it can be seen that taking HBCT test in the determination of material parameters into account, the good predictions of the Yeoh model based on UTT significantly deteriorated. This means that the Yeoh model may not be sufficient to model elastomeric bearings correctly in the whole range of deformation (in analyzed case bearings are compressed to the average value not crossing 7%). In the case of the neo-Hookean model, the discrepancies in the uniaxial tension test are even bigger (proper predictions up to extensions around 10% when only UTT was used for parameter determination and up to 40% when UTT and HBCT were used).
- The approach used in the article, taking into account the inverse method analysis and FEM modeling, is not automatic and requires verification at each stage (the influence of the FEM mesh, boundary conditions and local excessive deformation of finite elements at the steel–elastomer interface). Nevertheless, it allows us to extend the experimental base needed to determine material parameters in such a way that their application in problems with complex states of stresses, strains and deformations leads to rational results.
- Efficient modeling of elastomeric bearings even for engineering purposes (civil engineering) requires application of the large deformation theory and hyperelastic constitutive models. In such case, it is necessary to perform the UTT on the elastomer. This allows us, on one hand, to determine the scope of application of simplified models such as neo-Hookean, and on the other hand, it can be used to determine the input data for inverse method analysis and validation of more suitable hyperelasticity model.

## Figures and Tables

**Table 1:** The results of experimental results of UTT used to determine the material parameters of the hyperelasticity models.

$\mathrm{Elongation}\;\lambda_{1}(-)\;$	1.00	1.01	1.05	1.10	1.20	1.30	1.40	1.50	1.60	1.70	1.80	1.90	2.00	2.20	2.40
$\mathrm{Stress}\;S_{1}$ (MPa)	0.00	0.07	0.26	0.44	0.71	0.95	1.18	1.43	1.69	1.97	2.27	2.60	2.95	3.74	4.59

**Table 2:** Geometry of elastomeric bearings.

Height of the Bearing: LB, HB	H=41 (mm) , H=63 (mm)
Dimensions in plane	a × b=100 × 100 (mm ×mm)
Dimensions of reinforcing steel plates	$a^{\prime }\;\times \;b^{\prime }\;=\;90\;\times \;90\;(\mathrm{mm}\;\times \;$ mm)
Height of the inner layers of the elastomer	$h_{E}=8\;(\mathrm{mm})$
Height of the steel layers	$h_{S}=3\;(\mathrm{mm})$
Elastomer cover: top (bottom), side	2.5 (mm) , 5 (mm)
Number of reinforcing steel plates: LB; HB	4; 6

**Table 3:** The number of finite elements, nodes, vertical support reaction and analysis time depending on the mesh refinement.

	Number of Finite Elements	Number of Nodes	Vertical Reaction Force in Support	Error	Time of the Analysis *
	(-)	(-)	(kN)	(%)	(s)
Coarse mesh (M0)	20,095	26,002	−55.6	13.7	455
Medium mesh (M1)	63,007	55,263	−51.8	6.0	752
Detailed mesh (M2)	117,912	102,595	−50.8	3.9	1820
Very detailed mesh (M3)	312,809	280,902	−49.4	1.1	7724

* all calculations were performed on computer with four core processor i7 and 24 GB of RAM.

**Table 4:** Determined material parameters of constitutive models of elastomer.

	Model Parameters	Norm MSE
	(MPa)	(-)
neo-Hookean model parameters determined from the UTT	$C_{10}=0.8494$	0.1234
Yeoh model parameters determined from the UTT	$C_{10}=0.638\;$	0.0554
	$C_{20}=0.0430$	
	$C_{30}=0.00229$	
neo-Hookean model parameters determined from HBCT and UTT	$C_{10}=0.62$	0.0475
Yeoh model parameters determined from HBCT and UTT	$C_{10}=0.638$	0.0336
	$C_{20}=-0.0225$	
	$C_{30}=0.00161$	

## Data Availability

Data sharing is not applicable to this article.
